# A high spatial resolution dataset for methylmercury exposure in Guangdong-Hong Kong-Macao Greater Bay Area

**DOI:** 10.1038/s41597-023-02597-y

**Published:** 2023-10-17

**Authors:** Xiaoxin Zhang, Qiumeng Zhong, Weicen Chang, Hui Li, Sai Liang

**Affiliations:** 1 Key Laboratory for City Cluster Environmental Safety and Green Development of the Ministry of Education, School of Ecology, Environment and Resources, Guangdong University of Technology, Guangzhou, Guangdong 510006 China; 2 School of Environment, Beijing Normal University, Beijing, 100875 China

**Keywords:** Environmental impact, Environmental economics

## Abstract

Dietary methylmercury (MeHg) exposure increases the risk of many human diseases. The Guangdong-Hong Kong-Macao Greater Bay Area (GBA) is the world’s most populous bay area and people there might suffer a high risk of dietary MeHg exposure. However, there lacks a time-series high spatial resolution dataset for dietary MeHg exposure in the GBA. This study constructs a high spatial resolution (1 km × 1 km) dataset for dietary MeHg exposure in the GBA during 2009–2019. It first constructs the dietary MeHg exposure inventory for each county/district of the GBA, based on MeHg concentrations of foods (i.e., rice and fish in this study) and per capita rice and fish intake. Subsequently, this study spatializes the dietary MeHg exposure inventory at 1 km × 1 km scale, using gridded data for food consumption expenditure as the proxy. This dataset can describe the spatially explicit hotspots, distribution patterns, and variation trend of dietary MeHg exposure in the GBA. This dataset can support spatially explicit evaluation of MeHg-related health risks in the GBA.

## Background & Summary

Mercury (Hg) is a global persistent neurotoxic substance^1–3^. Inorganic mercury can be methylated into methylmercury (MeHg) with more toxicity by biochemical reaction^4^, ultimately harming human health. Dietary intake is the primary way of human exposure to MeHg^5^. Excessive and long-term low-dose MeHg intake poses a significant threat to human health, such as kidney and cardiovascular diseases^6^, psychological and neurological deficits^7^, and fetus intelligence quotient decrements^8^. Many countries have signed the Minamata Convention to protect human health from anthropogenic emissions and releases of Hg and Hg compounds^9^. Constructing a high spatial resolution dataset for dietary MeHg exposure can reveal spatially explicit hotspots of dietary MeHg exposure. This further helps to evaluate and reduce MeHg-related health risks.

The Guangdong-Hong Kong-Macao Greater Bay Area (GBA) is a world-class urban agglomeration. It consists of nine cities in the Pearl River Delta Area (including Guangzhou, Shenzhen, Zhuhai, Foshan, Huizhou, Dongguan, Zhongshan, Jiangmen, and Zhaoqing), Hong Kong Special Administrative Region (referred to as Hong Kong), and Macao Special Administrative Region (referred to as Macao). GBA is one of the regions with the most vigorous economic vitality in China and even the world^10^. The GBA consumes large amounts of rice and fish which are two primary pathways of dietary MeHg exposure^11,12^. Consequently, there is a potentially high risk of dietary MeHg exposure for the population of the GBA. Constructing a high spatial resolution dataset for dietary MeHg exposure in the GBA can lay the foundation for evaluating and reducing MeHg-related health risks of the GBA.

Existing studies on dietary MeHg exposure of the Chinese population mainly focus on the national and provincial scales. Little attention is paid to the high spatial resolution inventories of dietary MeHg exposure at the urban agglomeration (especially the GBA) scale. For example, Chen *et al*.^13^ compiled an inventory of dietary MeHg exposure for each province of China in 2010 and then revealed the related health risks. Li *et al*.^12^ constructed an inventory of dietary MeHg exposure via seafood consumption by different age groups in coastal areas of Guangdong. Chen *et al*.^14^ adopted the total diet study method to estimate the dietary MeHg exposure of the adult population in Hong Kong. For the Hg-related high spatial resolution datasets, Chang *et al*.^15^ have constructed a high spatial resolution dataset for anthropogenic atmospheric mercury emissions in China during 1998–2014 at a 1 km resolution. Li *et al*.^16^ mapped China’s MeHg-related health risks in 2010 at the 1 km × 1 km grid scale. However, a time-series high spatial resolution dataset for dietary MeHg exposure in the GBA is still insufficient. Such a time-series dataset can reveal the spatially explicit hotspots, distribution patterns, and variation trend of dietary MeHg exposure in the GBA.

This study aims to construct a gridded dataset for dietary MeHg exposure of the GBA with the 1 km × 1 km spatial resolution during 2009–2019. According to previous studies^17,18^, food types considered in this study include rice and fish (including freshwater and marine fish). This study first compiles a time-series inventory of dietary MeHg exposure for each county/district in the GBA during 2009–2019. Subsequently, it spatializes the dietary MeHg exposure in the GBA at the 1 km × 1 km scale, using gridded data on food consumption expenditure in the GBA as the proxy. This dataset can describe the spatially explicit hotspots, distribution patterns, and variation trend of dietary MeHg exposure in the GBA. It lays the foundation for evaluating and controlling MeHg-related health risks of the GBA.

## Methods

**Fig. 1:**
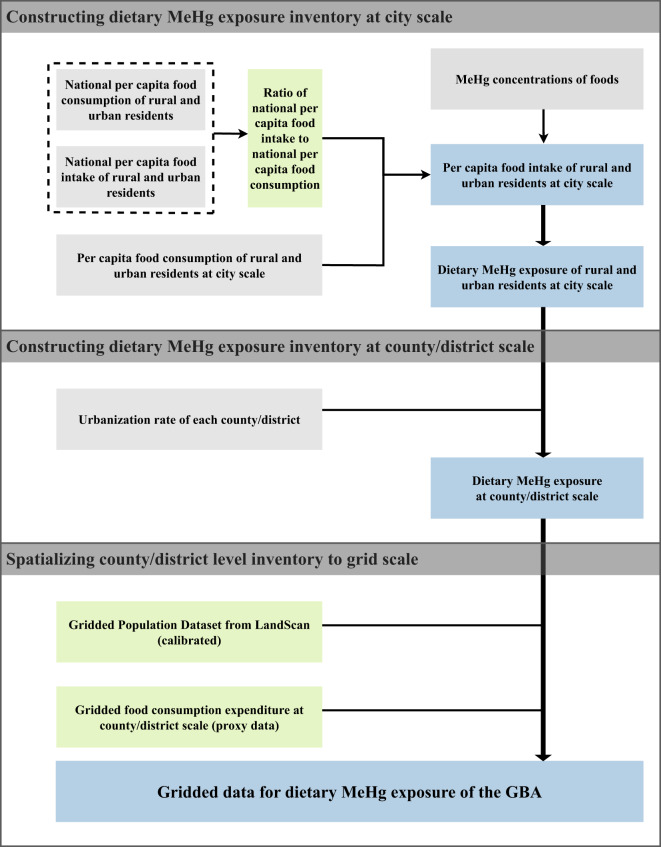
Procedures for constructing high spatial resolution dataset for dietary MeHg exposure in the GBA.

The construction of the time-series high spatial resolution dataset for dietary MeHg exposure in the GBA mainly consists of two steps (Fig. 1). We first construct the dietary MeHg exposure inventory for each county/district of the GBA, based on MeHg concentrations of foods (i.e., rice and fish in this study) and per capita rice and fish intake. Most counties/districts in this study are divided according to the current administrative divisions of the People’s Republic of China. It is worth noting that Guangming District, Longhua District, and Pingshan District of Shenzhen city were inaugurated late and there are few officially published data. According to the original affiliation of these three districts, Guangming District, Longhua District, and Baoan District are merged as Baoan District; Pingshan District and Longgang District are merged as Longgang District. Subsequently, we spatialize the dietary MeHg exposure inventory at 1 km × 1 km scale, using gridded data for food consumption expenditure of counties/districts as the proxy.

### Constructing dietary MeHg exposure inventory of counties/districts

Constructing dietary MeHg exposure inventory of counties/districts includes two steps: compiling MeHg concentrations of foods and evaluating estimated daily intake (EDI). According to previous studies^17,18^, food types considered in this study include rice and fish (including freshwater and marine fish).

#### Compiling MeHg concentrations of foods

This study collects the MeHg concentrations of rice and fish from published literature^19–31^ (see Supplementary Table 1). The data on MeHg concentrations of rice and fish for the GBA are scarce during 2009–2019. As an alternative, we use the MeHg concentration data for Guangdong province. Approximately 70% of the sampling sites for the concentration data we collected are located in the GBA. Thus, the concentration data we collected could represent the MeHg concentrations of foods in the GBA to some extent.

We collect the MeHg concentration data of each food type across different time points to reflect the temporal variation trends during 2009–2019. It is worth noting that the MeHg concentration data for each food type during 2009–2019 are discontinuous. For missing data in intermediate years, this study estimates them by an interpolation method. Taking the intermediate years between 2010 and 2014 as an example, the interpolation processes for 2011, 2012, and 2013 are as follows.

$$\begin{array}{ccc}2011\,{\rm{M}}{\rm{e}}{\rm{H}}{\rm{g}}\,{\rm{c}}{\rm{o}}{\rm{n}}{\rm{c}}{\rm{e}}{\rm{n}}{\rm{t}}{\rm{r}}{\rm{a}}{\rm{t}}{\rm{i}}{\rm{o}}{\rm{n}}\,{\rm{d}}{\rm{a}}{\rm{t}}{\rm{a}} & = & 2010\,{\rm{M}}{\rm{e}}{\rm{H}}{\rm{g}}\,{\rm{c}}{\rm{o}}{\rm{n}}{\rm{c}}{\rm{e}}{\rm{n}}{\rm{t}}{\rm{r}}{\rm{a}}{\rm{t}}{\rm{i}}{\rm{o}}{\rm{n}}\,{\rm{d}}{\rm{a}}{\rm{t}}{\rm{a}}+(2014\,{\rm{M}}{\rm{e}}{\rm{H}}{\rm{g}}\,{\rm{c}}{\rm{o}}{\rm{n}}{\rm{c}}{\rm{e}}{\rm{n}}{\rm{t}}{\rm{r}}{\rm{a}}{\rm{t}}{\rm{i}}{\rm{o}}{\rm{n}}\,{\rm{d}}{\rm{a}}{\rm{t}}{\rm{a}}\\  &  & -\,2010\,{\rm{M}}{\rm{e}}{\rm{H}}{\rm{g}}\,{\rm{c}}{\rm{o}}{\rm{n}}{\rm{c}}{\rm{e}}{\rm{n}}{\rm{t}}{\rm{r}}{\rm{a}}{\rm{t}}{\rm{i}}{\rm{o}}{\rm{n}}\,{\rm{d}}{\rm{a}}{\rm{t}}{\rm{a}})\times 0.25\end{array}$$

$$\begin{array}{ccc}2012\,{\rm{M}}{\rm{e}}{\rm{H}}{\rm{g}}\,{\rm{c}}{\rm{o}}{\rm{n}}{\rm{c}}{\rm{e}}{\rm{n}}{\rm{t}}{\rm{r}}{\rm{a}}{\rm{t}}{\rm{i}}{\rm{o}}{\rm{n}}\,{\rm{d}}{\rm{a}}{\rm{t}}{\rm{a}} & = & 2010\,{\rm{M}}{\rm{e}}{\rm{H}}{\rm{g}}\,{\rm{c}}{\rm{o}}{\rm{n}}{\rm{c}}{\rm{e}}{\rm{n}}{\rm{t}}{\rm{r}}{\rm{a}}{\rm{t}}{\rm{i}}{\rm{o}}{\rm{n}}\,{\rm{d}}{\rm{a}}{\rm{t}}{\rm{a}}+(2014\,{\rm{M}}{\rm{e}}{\rm{H}}{\rm{g}}\,{\rm{c}}{\rm{o}}{\rm{n}}{\rm{c}}{\rm{e}}{\rm{n}}{\rm{t}}{\rm{r}}{\rm{a}}{\rm{t}}{\rm{i}}{\rm{o}}{\rm{n}}\,{\rm{d}}{\rm{a}}{\rm{t}}{\rm{a}}\\  &  & -\,2010\,{\rm{M}}{\rm{e}}{\rm{H}}{\rm{g}}\,{\rm{c}}{\rm{o}}{\rm{n}}{\rm{c}}{\rm{e}}{\rm{n}}{\rm{t}}{\rm{r}}{\rm{a}}{\rm{t}}{\rm{i}}{\rm{o}}{\rm{n}}\,{\rm{d}}{\rm{a}}{\rm{t}}{\rm{a}})\times 0.5\end{array}$$

$$\begin{array}{ccc}2013\,{\rm{M}}{\rm{e}}{\rm{H}}{\rm{g}}\,{\rm{c}}{\rm{o}}{\rm{n}}{\rm{c}}{\rm{e}}{\rm{n}}{\rm{t}}{\rm{r}}{\rm{a}}{\rm{t}}{\rm{i}}{\rm{o}}{\rm{n}}\,{\rm{d}}{\rm{a}}{\rm{t}}{\rm{a}} & = & 2010\,{\rm{M}}{\rm{e}}{\rm{H}}{\rm{g}}\,{\rm{c}}{\rm{o}}{\rm{n}}{\rm{c}}{\rm{e}}{\rm{n}}{\rm{t}}{\rm{r}}{\rm{a}}{\rm{t}}{\rm{i}}{\rm{o}}{\rm{n}}\,{\rm{d}}{\rm{a}}{\rm{t}}{\rm{a}}+(2014\,{\rm{M}}{\rm{e}}{\rm{H}}{\rm{g}}\,{\rm{c}}{\rm{o}}{\rm{n}}{\rm{c}}{\rm{e}}{\rm{n}}{\rm{t}}{\rm{r}}{\rm{a}}{\rm{t}}{\rm{i}}{\rm{o}}{\rm{n}}\,{\rm{d}}{\rm{a}}{\rm{t}}{\rm{a}}\\  &  & -\,2010\,{\rm{M}}{\rm{e}}{\rm{H}}{\rm{g}}\,{\rm{c}}{\rm{o}}{\rm{n}}{\rm{c}}{\rm{e}}{\rm{n}}{\rm{t}}{\rm{r}}{\rm{a}}{\rm{t}}{\rm{i}}{\rm{o}}{\rm{n}}\,{\rm{d}}{\rm{a}}{\rm{t}}{\rm{a}})\times 0.75\end{array}$$

For the other missing data, this study estimates them based on the assumption that the changing rates in adjacent years are the same. If there are two or more values for MeHg concentration of a food in the same year, this study takes the average as the MeHg concentration of this food in that year.

#### Evaluating estimated daily intake (EDI)

Calculating the EDI requires the data on per capita intakes of foods and MeHg concentrations of foods. The data on per capita intakes of foods at the county/district level are unavailable from existing statistics. We assume that the per capita intakes of foods by urban and rural residents in each county/district are, respectively, the same as those in the corresponding city. Consequently, we can calculate the EDI of MeHg by residents in each county/district based on the EDIs of MeHg by urban and rural residents at the city level and the urbanization rate of each county/district.

Firstly, we evaluate the EDIs for urban and rural residents at the city scale by multiplying the municipal per capita intakes of foods with MeHg concentrations of foods. The per capita intakes of foods in each city of the GBA can be derived from the data on per capita food consumption. Per capita food consumption is close to per capita food intake, but they are different. We use the ratio of national per capita food intake to national per capita food consumption to derive the per capita food intake in each city of the GBA, based on the per capita food consumption in each city of the GBA. The national per capita food intake of rural and urban residents can be obtained from the China Health and Nutrition Survey (CHNS)^32^. Most city level and national data on per capita food consumption (kg*year^−1^*capita^−1^) of rural and urban residents can be obtained from City Statistical Yearbooks^33–41^ and China Statistical Yearbook^42^, respectively. The data for per capita food consumption in Hong Kong can be obtained from the Hong Kong Population-based Food Consumption Survey^43,44^. For the missing data of certain cities (e.g., Jiangmen and Zhongshan), we use the average level of cities with similar per capita GDP. In particular, among the GBA, the per capita GDP of Macao is close to that of Hong Kong. The dietary pattern of Macao’s residents is close to that of the Pearl River Delta^45^ and Zhuhai is adjacent to Macao. Therefore, we use the per capita food consumption of Hong Kong and dietary structure of Zhuhai to calculate the per capita food consumption of Macao.

Meanwhile, for certain years, food categories of the physical per capita food consumption data are highly aggregated. We disaggregate the data by two methods. Preferentially, we divide the monetary per capita consumption expenditure of particular food products (Yuan year^−1^ capita^−1^) by corresponding consumer prices. Alternatively, we disaggregate the data according to the proportions of food production. The data for monetary per capita food consumption expenditure, consumer prices, and food production can be obtained from the yearbooks mentioned above.

The EDI at the city level is calculated with Eq. (1) and Eq. (2).

1 $${I}_{i,j}=CO{N}_{i,j}\times \frac{N{I}_{i}}{NCO{N}_{i}}$$

2 $$ED{I}_{j}=\sum _{i}\left({I}_{i,j}\times {C}_{i}\right)/BW$$

The notation I_i,j_ indicates per capita intake (g d^−1^ capita^−1^) of food *i* in city *j* and CON_i,j_ indicates per capita consumption (g d^−1^ capita^−1^) of food *i* in city *j*. The noataions NI_i_ and NCON_i_ represent national per capita intake and per capita consumption of food *i* in China, respectively. The notation EDI_j_ denotes the EDI of MeHg in city *j*; C_i_ represents the MeHg concentration of food *i* in Guangdong; and BW means the average weights of Chinese adult males (66.2 kg) and females (57.3 kg)^46^.

Secondly, we downscale the EDI of MeHg by residents from the city scale to the county/district scale, based on the EDIs of MeHg by urban and rural residents at the city level and the urbanization rate of each county/district. The EDI at the county/district scale is calculated with Eq. (3).

3 $$ED{I}_{n}=UED{I}_{j}\times r+RED{I}_{j}\times \left(1-r\right)$$

The notation EDI_n_ denotes the EDI of MeHg for county/district *n* in city *j*; UEDI_j_ and REDI_j_ indicate the EDIs of MeHg by urban and rural residents in city *j*, respectively; and *r* refers to the urbanization rate of county/district *n*.

### Spatializing county/district level inventory to grid scale

We use the gridded data on food consumption expenditure of counties/districts in the GBA as proxy data to spatialize the dietary MeHg exposure (i.e., EDI of MeHg) of counties/districts. In this way, we can construct a high spatial resolution dataset for dietary MeHg exposure in the GBA. Here we take 2010 as an example to explain the spatializing procedures.

1. We obtain and calibrate the gridded population data in the GBA. The gridded population data are from the LandScan population dataset developed by Oak Ridge National Laboratory^47^, covering the Pearl River Delta in Guangdong Province, Hong Kong, and Macao during 2009–2019. In this study, we assign zero to the gridded population where the value is null. It is worth noting that the total population of each county/district in the gridded population dataset is inconsistent with that of official statistics. We hence calibrate the gridded population dataset for each county/district according to the total population data in City Statistical Yearbooks, as shown in Eq. (4).
  4 $${p}_{n,i}^{2010}={g}_{n,i}^{2010}\times \frac{po{p}_{n}^{2010}}{{\sum }_{i}{g}_{n,i}^{2010}}$$
  The notations p_n,i_^2010^ and pop_n_^2010^ denote the population of grid *i* in county/district *n* (after calibration) and the population of county/district *n* from official statistics in 2010, respectively. The notation g_n,i_^2010^ represents the population of grid *i* in county/district *n* in 2010 from gridded population dataset before calibration.
2. We multiply the per capita food consumption expenditure of counties/districts by gridded population to obtain the gridded data for food consumption. The data for per capita food consumption expenditure of counties/districts are unavailable from official statistics. One way to estimate per capita food consumption expenditure of counties/districts is multiplying the total per capita consumption expenditure of each county/district with Engel’s coefficient. Another way is calculating the ratio of per capita food consumption expenditure to per capita disposable income in each city and then using this ratio to adjust per capita disposable income of each county/district. These data can be obtained from City Statistical Yearbooks and County/District Statistical Yearbooks.
  The gridded food consumption expenditure of the GBA in 2010 is calculated with Eq. (5).
  5 $$CO{N}_{n,i}^{2010}={p}_{n,i}^{2010}\times pCO{N}_{n,i}^{2010}$$
  The notation CON_n,i_^2010^ represents the food consumption expenditure in grid *i* of county/district *n* in the GBA and pCON_n_^2010^ denotes the per capita food consumption expenditure of county/district *n* in 2010.
3. Consequently, the dietary MeHg exposure in each grid of the GBA is calculated with Eq. (6).

6 $$ED{I}_{n,i}^{2010}=ED{I}_{n}^{2010}\times \frac{CO{N}_{n,i}^{2010}}{{\sum }_{i}CO{N}_{n,i}^{2010}}$$

The notation EDI_n,i_^2010^ denotes the EDI of MeHg in grid *i* of county/district *n* and EDI_n_^2010^ represents the total EDI of MeHg in county/district *n* in 2010.

## Data Records

This dataset is publicly available under Zenodo^48^. Its values are in μg kg^−1^ d^−1^. The GBA consists of the Pearl River Delta, Hong Kong, and Macao, and we hence classify the gridded data on dietary MeHg exposure into three parts (i.e., the Pearl River Delta, Hong Kong, and Macao). The data files are in tif format and named based on the years. Table 1 shows the naming protocol for this dataset.

**Table 1:** Naming method of data files in the database.

Regions	File naming
Pearl River Delta	2009.tif - 2019.tif
Hong Kong	2009.tif - 2019.tif
Macao	2009.tif - 2019.tif

## Technical Validation

This study uses the methods from peer reviewed literature to construct the gridded dataset for dietary MeHg exposure in the GBA. For example, the methods for calculating the per capita intake and EDI are from Chen *et al*.^13^ The method to spatialize the dietary MeHg exposure is from Li *et al*.^16^. However, there are still uncertainties in the dataset of this study, mostly from the assumptions of calculation methods.

1. When data for counties/districts are unavailable, we calculate them by referring to parameters of corresponding cities or Guangdong Province. This assumption would lead to uncertainties of the results. For example, the data for per capita food consumption of counties/districts in the GBA are unavailable from official statistics, and hence the EDI of MeHg at the county/district level cannot be obtained directly. We referred to the EDIs of MeHg by urban and rural residents at the city level in the calculations. This assumption would result in uncertainties in the estimated EDI of MeHg at the county/district level.
2. The processing of the gridded population data would also lead to uncertainties. For areas with extremely low population density (e.g., areas close to the coast and mountains or areas with large landscapes), their gridded population values are null. We assume that the population density of these areas is zero. Thus, ‘zeros processing’ is performed for the data with null values.
3. For Hong Kong and Macao, their population data from official statistics are temporally discontinuous. This study uses the interpolation method to estimate the missing values for intermediate years.

We conducted 10,000 Monte Carlo simulations of the variables in the construction of the MeHg intake inventory, and the statistical distributions of the P10 and P90 values are set as the lower and upper limits of the uncertainty range. Detailed uncertainty results for the EDI of MeHg are shown in Supplementary Table 2. The uncertainties of this dataset can be reduced through three aspects. (1) Improving the statistical system of countries/districts in the GBA could reduce the data unavailability. This can avoid parts of the assumptions and related uncertainty during the calculation of the EDI of MeHg at the county/district level. (2) Improving the accuracy of the proxy gridded data (i.e., to be more consistent with official statistics) can also reduce the uncertainty of gridded dietary MeHg exposure in the GBA. (3) For the estimation of missing data in certain years, more advanced methods (e.g., machine learning and artificial intelligence) can be used to complement the interpolation method. The combination of these methods is a promising way to reduce the uncertainty of estimated data.

To evaluate the rationality of our dataset, we compare our results of dietary MeHg exposure with published data (Table 2). Liu *et al*.^17^ analyzed China’s average dietary MeHg exposure in 2011. Our result in the GBA is similar to that of Liu *et al*. However, the result of this study is much different from that of Shao *et al*.^11^ in the Pearl River Delta. There are mainly two reasons. (1) The food scope is different. Existing studies regard rice and fish to be food sources of dietary MeHg exposure^17^,^18^. Thus, this study only considers rice and fish. Other food types in Liu *et al*.^17^ and Shao *et al*.^11^ are not included. (2) The quality and sources of data are different. The data of our study and Liu *et al*.^17^ are from official statistics (CHNS with large samples) and peer reviewed literature. Shao *et al*.^11^ conducted a questionnaire survey with relatively small samples. Moreover, about half of the survey areas in the study of Shao *et al*.^11^ are located in fishing villages with relatively high fish consumption.

**Table 2:** Comparisons of our dataset and published literature.

Literature	Years	Food types	Regions	Data sources	Dietary MeHg exposure (μg kg^−1^ d^−1^)
Shao *et al*.^11^	—	Cereal, vegetables, meat, and fish	Pearl River Delta	Questionnaire survey (*n* = 91)	0.215
Liu *et al*.^17^	2011	Rice, beans, vegetables, pork, poultry, eggs, and fish	China	Official statistics (CHNS) and literature	0.057
This dataset	2009–2019	Rice and fish	Greater Bay Area	Official statistics (CHNS) and literature	0.049 (2011)

### Supplementary information

Supplementary Table 1

Supplementary Table 2

## Data Availability

The processing of gridded data is carried out in ArcGis10.6. The dataset for dietary MeHg exposure in the GBA at 1 km × 1 km scale is available in the open-access online dataset Zenodo^48^.
